# A multi-peak performance landscape for scale biting in an adaptive radiation of pupfishes

**DOI:** 10.1242/jeb.247615

**Published:** 2024-08-23

**Authors:** Anson Tan, Michelle St. John, Dylan Chau, Chloe Clair, HoWan Chan, Roi Holzman, Christopher H. Martin

**Affiliations:** ^1^ Department of Integrative Biology, University of California, Berkeley, Berkeley, CA 94720-3140, USA; ^2^ Museum of Vertebrate Zoology, University of California, Berkeley, Berkeley, CA 94720, USA; ^3^ Department of Biology, University of Oklahoma, Norman, OK 73019, USA; ^4^ Department of BioSciences, Rice University, Houston, TX 77005, USA; ^5^ School of Zoology, Wise Faculty of Life Sciences, Tel Aviv University, Ramat Aviv 69978, Israel; ^6^ Inter-University Institute for Marine Sciences, Eilat 8810302, Israel

**Keywords:** Adaptive radiation, Functional morphology, Ecomorphology, Bite kinematics, Lepidophagy, Trophic specialization, Performance landscape

## Abstract

The physical interactions between organisms and their environment ultimately shape diversification rates, but the contributions of biomechanics to evolutionary divergence are frequently overlooked. Here, we estimated a performance landscape for biting in an adaptive radiation of *Cyprinodon* pupfishes, including scale-biting and molluscivore specialists, and compared performance peaks with previous estimates of the fitness landscape in this system. We used high-speed video to film feeding strikes on gelatin cubes by scale eater, molluscivore, generalist and hybrid pupfishes and measured bite dimensions. We then measured five kinematic variables from 227 strikes using the SLEAP machine-learning model. We found a complex performance landscape with two distinct peaks best predicted gel-biting performance, corresponding to a significant non-linear interaction between peak gape and peak jaw protrusion. Only scale eaters and their hybrids were able to perform strikes within the highest performance peak, characterized by larger peak gapes and greater jaw protrusion. A performance valley separated this peak from a lower performance peak accessible to all species, characterized by smaller peak gapes and less jaw protrusion. However, most individuals exhibited substantial variation in strike kinematics and species could not be reliably distinguished by their strikes, indicating many-to-many mapping of morphology to performance. The two performance peaks observed in the lab were partially consistent with estimates of a two-peak fitness landscape measured in the wild, with the exception of the new performance peak for scale eaters. We thus reveal a new bimodal non-linear biomechanical model that connects morphology to performance to fitness in a sympatric radiation of trophic niche specialists.

## INTRODUCTION

The major process generating new biodiversity is adaptive radiation, in which a single lineage colonizes myriad ecological niches and rapidly diversifies (Gillespie et al., 2020; Martin and Richards, 2019; Simpson, 1944; Stroud and Losos, 2016). However, this process of rapid adaptation is also constrained by physical laws governing interactions between the organism and the essential tasks needed for survival and reproduction within its environment, known as the emerging field of ecomechanics (Higham et al., 2016, 2021; Perevolotsky et al., 2020). Understanding how functional traits interact in complex ways to achieve these essential life history tasks is fundamental for connecting phenotypes to performance to fitness to adaptive radiation within new environments. These relationships can be visualized as a performance landscape (Arnold, 1983, 2003; Martin et al., 2019; Simon and Moen, 2023). For example, estimating the performance landscape for a functional task or tasks can lead to deep insights about patterns of morphospace occupation and rates of morphological diversification across diverse taxa (Armbruster, 1990; Benkman, 1993; Figueirido et al., 2013; Holzman et al., 2022; Olsson et al., 2020; Raup, 1966; Schultz et al., 2021; Stayton, 2019; Tseng and Flynn, 2018). However, very few systems have been investigated from the perspectives of both performance and fitness landscapes to examine how congruence and many-to-many mapping between these high-dimensional landscapes may promote or constrain evolutionary diversification (e.g. Beausoleil et al., 2023; Calsbeek and Irschick, 2007; Ibáñez de Aldecoa et al., 2024; Stroud et al., 2023).

Scale feeding, or lepidophagy, is a fascinating ecological niche that has evolved at least 20 times in fishes across a diverse range of environments (Martin and Wainwright, 2013c), from rift lakes (Hori, 1993; Raffini et al., 2017; Stewart and Albertson, 2010; Takahashi et al., 2007) to tropical streams and rivers (Evans et al., 2017; Gosavi et al., 2018, 2019; Grubh et al., 2004) to the mesopelagic zone (Nakae and Sasaki, 2002); and across ontogenetic stages (Davis et al., 2011; Kolmann et al., 2018; Novakowski et al., 2004; Szelistowski, 1989). Scale feeding has also evolved in a diverse range of fish groups, including Cichliformes, Characiformes, Siluriformes, Tetraodontiformes and Squaliformes (Kolmann et al., 2018; Martin and Wainwright, 2013c; Nakae and Sasaki, 2002; Sazima, 1984). In contrast to piscivory, all scale eaters are generally smaller than their larger prey and numerous strikes must be performed efficiently because of the low energy payoff in calories per strike, resulting in an excellent laboratory and field model for observing repeated prey capture behaviors. Scale eaters rarely or never consume whole prey (Gosavi et al., 2018; Kovac et al., 2019; Martin and Wainwright, 2013c; Sazima, 1984, 1986). Therefore, this trophic niche provides an excellent setting for investigating the functional traits underpinning performance and fitness because successful scale eating appears to require both intensive prey capture behaviors and efficient energy use per strike (Sazima, 1984).

The scale-eating pupfish, *Cyprinodon desquamator*, is the youngest lepidophagous species known, as it evolved only ∼10–15 kya on San Salvador Island, Bahamas (Martin and Wainwright, 2013a; Martin et al., 2019). Scales comprise approximately 50% of its diet in addition to macroalgae and microinvertebrates in the several hypersaline lakes where it is endemic (Martin and Wainwright, 2011, 2013c; McLean and Lonzarich, 2017). All other extant scale-eating lineages likely evolved at least 1 Mya (Koblmüller et al., 2007), except for the extinct Lake Victorian lepidophagous cichlid *Haplochromis welcommei* (Greenwood, 1965). In several interior hypersaline lakes on San Salvador Island, *C. desquamator* occurs in sympatry at low frequencies in the same benthic macroalgae-dominated habitats as *Cyprinodon variegatus*, a widespread generalist species, *Cyprinodon brontotheroides*, an endemic oral-shelling molluscivore species, and *Cyprinodon* sp. ‘wide-mouth’, a newly discovered intermediate scale-eating ecomorph, which was not included in this study (Hernandez et al., 2018; Martin and Feinstein, 2014; Richards and Martin, 2022; St John et al., 2020a,b).

The adaptive radiation on San Salvador Island exhibits one of the fastest rates of craniofacial diversification among any measured vertebrate group, up to 1000 times faster than generalist populations on neighboring Bahamian islands for oral jaw length, as a result of rapid adaptation to the novel trophic niche of lepidophagy (Martin, 2016b; Martin and Wainwright, 2011). Previous field experiments measuring the growth and survival of F2 and F5 hybrids among all three species placed in field enclosures within two hypersaline lakes on San Salvador Island estimated a two-peak fitness landscape and a large fitness valley isolating the hybrids with greatest morphological similarity to scale eaters (Martin and Wainwright, 2013b; Patton et al., 2022). This two-peak fitness landscape was stable over multiple years, lake environments and phenotype–frequency manipulations, suggesting that biophysical constraints on the interaction between pupfish craniofacial traits and their bite performance may ultimately shape the adaptive landscape in this system, rather than frequency-dependent competition (e.g. Hori, 1993; Martin, 2012) or seasonal resource abundance (Grant and Grant, 2002), which would predict a dynamically changing landscape (Martin, 2016a; Martin and Gould, 2020). In a previous laboratory study of scale biting, we compared strike kinematics across all three species of San Salvador Island pupfish while they consumed shrimp, scales and gelatin cubes in the lab (St John et al., 2020a,b). We found that scale-eating pupfish exhibited peak gapes that were twice as large as those of other species and attacked prey with a more obtuse angle between their lower jaw and suspensorium, resulting in more surface area removed per strike (St John et al., 2020a,b). However, performance landscapes have not previously been estimated in this system to allow for direct comparison to fitness landscapes.

Our goal in this study was to estimate the performance landscape for scale biting. We hypothesized that the performance landscape resulting from physical interactions between divergent craniofacial morphology and bite kinematics should correspond to features of the fitness landscape. The degree of correspondence between the location of performance and fitness optima should indicate the extent to which this particular functional task is relevant to the growth and survival of these species in the wild as measured in our previous studies on this system (reviewed in Arnold, 2003; Arnold et al., 2001; Hansen et al., 2012; Martin et al., 2019; Simon and Moen, 2023). We compared the correspondence between the fitness landscape in the wild (growth and survival of different hybrid morphologies) with the performance landscape of each species and their hybrids in laboratory gel-biting kinematic trials. We sampled extensive phenotypic diversity within the radiation using both purebred species and multiple F2 hybrid intercrosses and backcrosses to characterize kinematic performance across the entire morphospace. We also developed a new automated machine-learning pipeline that enabled us to accelerate the rate of data acquisition, one of the major bottlenecks for kinematic analyses. We predicted that fitness peaks observed for generalist and molluscivore morphology in the wild would correspond to performance peaks for these two species in the lab, plus a third performance peak for scale biting that was not detected in field experiments.

## MATERIALS AND METHODS

### Collection and husbandry

Using seine nets or hand nets, we collected molluscivore (*Cyprinodon brontotheroides* Martin & Wainwright 2013) and scale-eating (*Cyprinodon desquamator* Martin & Wainwright 2013) pupfish from Crescent Pond, Little Lake and Osprey Lake on San Salvador Island, Bahamas, and generalist (*Cyprinodon variegatus* Lacepède 1803) pupfish from Lake Cunningham in New Providence Island, Bahamas, and Fort Fisher estuary in North Carolina, USA, in 2017 and 2018. Generalist pupfish from San Salvador Island could not be readily trained to feed on gelatin cubes during the period of this study; however, generalists from neighboring islands exhibit essentially identical craniofacial morphology and kinematics relative to San Salvador Island generalists (Martin, 2016b; St John et al., 2020a,b). Wild-caught and lab-reared fish were maintained in 40–80l aquaria at salinities of 2–8 ppt and 23–27°C and fed a diet of frozen bloodworms, frozen mysis shrimp and commercial pellet foods daily. This study used only second to fourth generation lab-reared pupfishes. All San Salvador Island species can be readily crossed to produce viable and fertile hybrids (Martin et al., 2017; St John et al., 2024). F1 hybrid and F2 hybrid intercrosses were generated from molluscivore×scale eater crosses from both Osprey Lake and Crescent Pond, generalist×scale eater and generalist×molluscivore crosses from Little Lake, and generalist×*Cyprinodon* sp. ‘wide-mouth’ F1 hybrids from Osprey Lake (Richards and Martin, 2022). All newly hatched fry were fed *Artemia* nauplii for approximately 1 month. Prior to filming, pupfishes were fed exclusively Repashy Superfood gel diets for acclimation and training for at least 1 week before filming. All animal procedures used in this study were approved by the UC Berkeley Animal Care and Use Committee (AUP-2021-07-14515, AUP-2021-02-14062-1, AUP-2020-02-12986, AUP-2018-08-11373).

### High-speed filming and measurement of gelatin bites

We recorded pupfishes feeding on standardized gelatin cubes (dimensions: 1.5 cm×1.5 cm×1.5 cm; Community Plus Omnivore Gel Premix, Repashy Specialty Pet Products). Gels were prepared in batches of 50 at precisely a 4:1 water:mix ratio in silicone molds following the manufacturer's instructions and allowed to set overnight at 4°C. Gels were stored covered at 4°C for a maximum of 2 weeks before discarding. The gel cube retains its shape in water and therefore allows precise measurement of the dimensions and area removed by each bite.

**Fig. 1.**
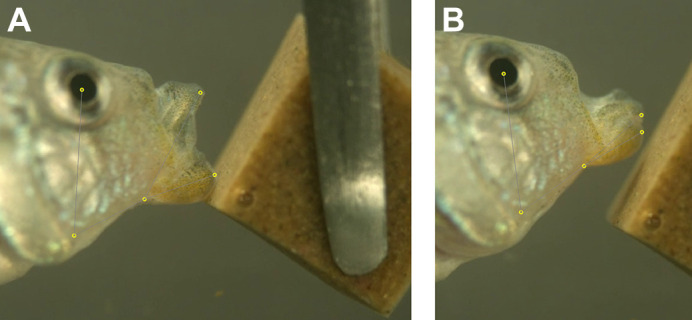
**Digitizing high-speed video of gel-biting strikes with automated landmarking software.** Strike sequences for a hybrid pupfish strike at peak gape (A) and a miss (B). Videos were filmed at 1100 frames s^−1^ on a Phantom VEO 440S camera. Five landmarks on each frame were placed automatically using our SLEAP inference model and are illustrated as small yellow dots to emphasize the accuracy of these inferred landmarks (extended version in Fig. S2; Automated landmarking of video sequences is provided in Movies 1–3).

Individuals were trained in group tanks to feed on gelatin cubes and then netted individually for filming. We filmed all strikes at 1100 frames s^−1^ using a full-color Phantom VEO 440S (Vision Research Inc.) fitted with a Canon EF-S 60 mm lens mounted on a standard tripod. Fish were filmed individually in 7.5 l bare glass tanks with a solid matte background at salinities of 2–3 ppt and 21–23°C. Illumination was provided by two dimmable full-spectrum LED lights placed on either side of the filming tank. Gelatin cubes were held with forceps with one edge facing in a horizontal direction toward the fish (Fig. 1). Trained fish usually attacked the gel almost immediately after placement in the filming tank. After each strike, the cube was immediately removed and inspected to confirm a bite mark; missed strikes were confirmed from the video replay. New cubes were used for each feeding strike and never re-used. All videos were filmed in lateral view. In total, 37 individuals were filmed performing 227 strikes that were successfully scored for all landmarks using automated tracking software. Of these, we were able to measure a subset of 132 strikes with available data for bite dimensions from gelatin cubes (Table 1). Strikes per individual ranged from 1 to 10 (Table 1). In some cases (14 of the 130), fish made contact with the gel but left no bite marks (hereafter ‘missed strikes’). Missing bite data were due to accidentally damaged or crushed gels and early pilot videos for which bite dimensions were not recorded. Strikes in which the jaws did not make visible contact with the gel were excluded.

**Table 1.**
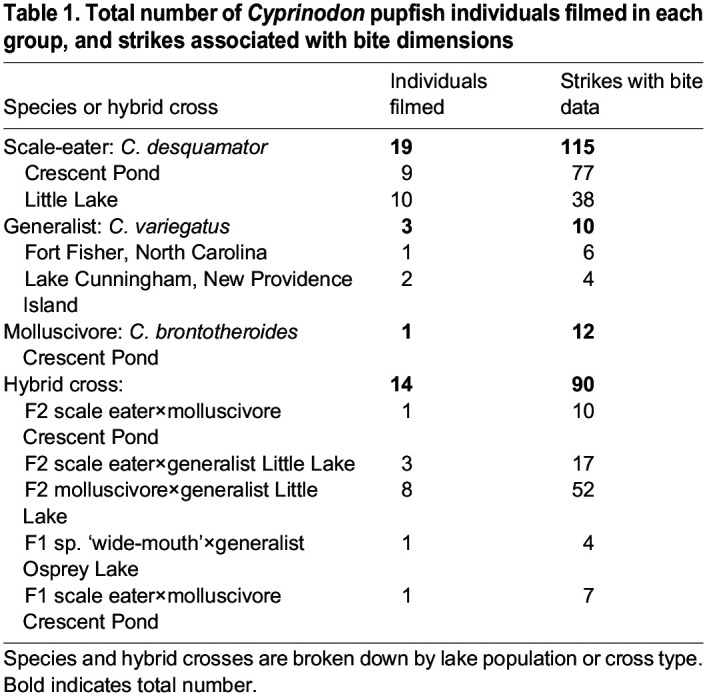
Total number of *Cyprinodon* pupfish individuals filmed in each group, and strikes associated with bite dimensions

**Fig. 2.**
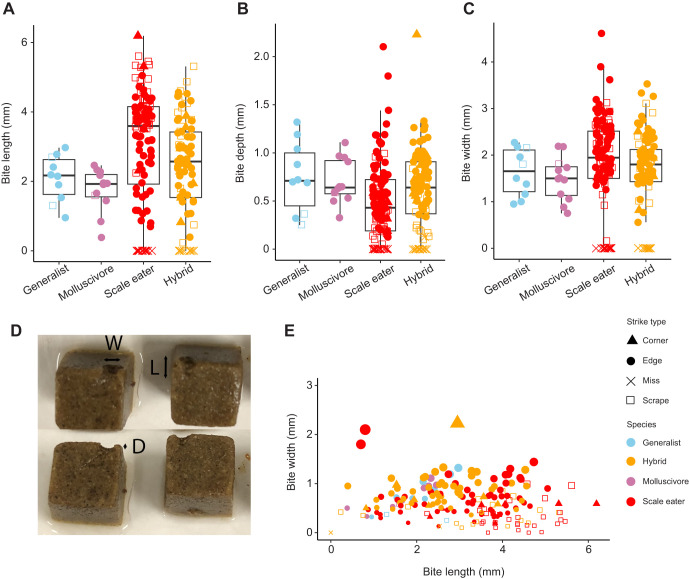
**Bite dimensions.** (A–C) Boxplots (median, upper and lower quartiles and 1.5× interquartile range) overlaid with raw data show the three bite dimensions (A, length; B, depth; C, width) across each species or hybrid cross. Strike type is indicated by symbol shape. (D) Top-down and side views of typical bites from Repashy gelatin cubes following a single strike. Bite dimensions (L, length; D, depth; W, width) are labeled and were measured immediately after each strike using digital calipers under a stereomicroscope. (E) Scatterplot of bite width by depth with species and strike type indicated by symbol color and shape, respectively. The relative size of each point is proportional to the depth of the bite.

The length, width and depth of each gelatin bite were measured using digital high-precision calipers (Mitutoyo) under a stereomicroscope (Fig. 2). Strikes were characterized as an edge, corner, scrape or miss. Edge bites occurred along the edge but not the corner of the gelatin cubes. Corner bites, defined by chunks removed from one of the corners, were distinguished from edge strikes because bite orientation may affect kinematic performance and bite dimensions. Scrapes were defined by bites in which the jaws did not completely close around the gel to remove a chunk of the material, but instead left two distinct indentations. Misses were defined as strikes in which the oral jaws of the fish contacted the gel but did not leave any marks. Bites were approximately elliptical in shape (Fig. 2) and length was the longest axis of the bite, spanning across two faces of the gel for edge and corner bites or one face only for scrapes. Volume was calculated from the length×width×depth of each bite and likely varied by strike type; therefore, we included the effects of strike type in all models and additionally included bite length and depth as a performance metric. Bite width was similar across strikes for each individual and largely reflected an individual's gape width, which is morphologically constrained and does not vary much during feeding strikes. All individuals were filmed consecutively over one or two filming periods for up to 16 strikes. After each filming session for each individual, an image of a ruler was photographed in the filming tank at the same distance as the gelatin cubes for calibration of videos.

### Machine learning for quantifying kinematic landmarks on each frame

We used the SLEAP (Social LEAP Estimates Animal Poses) animal tracking software to automatically detect and place morphometric landmarks on each frame (Pereira et al., 2020, 2022). This software supports data input of raw videos and then provides an interactive graphical user interface (GUI) to create a labeled training dataset. Predictions from trained models can then be adjusted to enable a ‘human-in-the-loop’ workflow to efficiently and progressively obtain more accurate models and inferences of landmarks. To train the model, we manually placed five landmarks on 815 frames using the SLEAP GUI. These training frames spanned 100 high-speed feeding videos including all three species and their hybrids. Frames were chosen for labeling both by the users and automatically by the software to span highly divergent scenes spread across the beginning, middle and end of each strike. Landmarks included the anterior-most tip of the upper and lower jaw, the center of the pupil, the posteroventral margin of the opercle, and the posterior-most visible point of the lower jaw as a proxy for the lower jaw joint.

We trained a model based on the labeled data after exploring various configurations. We achieved the greatest number of landmarked videos and frames using the multi-animal bottom-up u-net model with a receptive field of 156 pixels, maximum stride of 32 pixels, batch size of 3, input scaling of 0.75, and validation fraction of 0.1 (SLEAP model error is plotted in Fig. S1). We trained this model on a laptop running a 16 Gb NVIDIA GeForce 3070 GPU, which completed training in less than 12 h. We then used this trained model to infer landmarks on each frame of a larger set of strike videos using the flow cross-frame identity tracker, which shares information about landmark locations across frames for each individual strike video. We culled to a single instance (i.e. one animal) per frame, given that fish were filmed individually, and connected single track breaks. This automated procedure predicted landmarks on 114,000 frames from 227 videos (Fig. 1; Fig. S1). Pixel sizes of each landmark varied across videos based on the zoom level and relative size of each fish; however, inferred landmarks generally could not be distinguished from human landmarks in our final model (Movies 1–3; Fig. 1). Coordinate data from each frame were exported in .hdf5 format, imported into R (http://www.R-project.org/), and stored in any array using the rhdf5 package (https://github.com/grimbough/rhdf5). R code is provided in Dataset 2.

### Kinematic variables

We characterized gel-biting behavior based on five kinematic variables deemed important for scale biting (St John et al., 2020a,b). (1) Peak gape, the distance from the anterior tip of the premaxilla to the anterior tip of the dentary. (2) Peak jaw protrusion, the distance from the center of the pupil to the anterior tip of the premaxilla. (3) Peak lower jaw angle, the minimum angle between the lower jaw, the jaw joint and the ventral surface of the fish beneath the suspensorium measured from an anteroventral landmark on the preopercle. This measures the maximum rotation of the oral jaws in a downward and outward direction toward the gel as defined in St John et al. (2020a,b). Note that in Cyprinodontiformes, oral jaw opening is decoupled from jaw protrusion by the maxillomandibular ligament such that peak gape does not necessarily occur simultaneously with peak jaw protrusion (Hernandez et al., 2009). (4) Time to peak gape (TTPG; ms), the time from 20% of peak gape to peak gape. (5) Ram speed (m s^−1^), calculated as the distance traveled by the fish (measured at the center of the eye in mm) from the time at 20% of peak gape to the time at peak gape divided by the time to peak gape (ms). Milliseconds were calculated by counting frames and multiplying by 0.909 (1/1100 frame rate per second).

### Statistical analyses

#### Mixed-effect modeling

Our first step was to test the hypothesis that species and their hybrids differed in their feeding kinematics and performance. Because of our repeated measures design of multiple strikes per fish, we used mixed-effects models to compare kinematic variables and bite dimensions across species groups. We used the lme4 and lmerTest packages in R to fit linear mixed-effects models for each of the five kinematic response variables as well as bite dimensions with independent fixed effects for strike type (edge, corner, scrape or miss) and species (scale eater, molluscivore, generalist or hybrid) plus the random intercept effect of individual ID. We used the normal distribution to model each kinematic response variable and checked for normality of residuals from each model using quantile–quantile plots in R with the qqnorm command for linear mixed-effect models (Pinheiro and Bates, 2000). *P*-values were assessed for each factor level using Satterthwaite's method, which accounts for unequal variances across groups (Kuznetsova et al., 2017). We used the emmeans package in R to run *post hoc* tests for specific comparisons of interest between factor levels (Searle et al., 1980). We used Akaike's information criterion (AIC) to compare additional models with random slopes and interactions among the fixed effects (Burnham et al., 2011).

We did not correct for phylogenetic signal in our analyses because of the failure of this radiation to fit a tree-like model of evolution as a result of extensive secondary gene flow (Richards and Martin, 2017; Richards et al., 2021), in addition to our inclusion of several hybrid crosses. However, we note that both generalist outgroup populations are more distantly related to each other than to the scale eater, molluscivores and hybrids on San Salvador Islands (Martin and Feinstein, 2014), indicating that kinematic variables and bite dimensions exhibit minimal phylogenetic signal (Losos, 2008).

#### Multivariate analyses of kinematic variation

We used linear discriminant analysis from the MASS package in R (Ripley et al., 2013; https://CRAN.R-project.org/package=MASS) to assess overall discrimination among species and strike type based on variation in the five kinematic variables. We further calculated classification accuracy using species or strike type as the grouping variable using all five kinematic variables.

#### Generalized additive modeling

We used generalized additive semi-parametric models (GAMs) to reconstruct the performance landscape, i.e. the expected performance under each combination of phenotypic (kinematic) traits (Holzman et al., 2022). We used GAMs as they allow for non-linear terms and interactions between kinematic variables and bite dimensions (Wood and Augustin, 2002). Because we were interested in directly predicting bite performance from the kinematics data for each strike, we treated strike as our unit of replication, not fish, and did not control for individual in our statistical models. However, within-individual variation often exceeded between-species variation and we were not directly interested in species kinematic differences using this modeling framework, which we previously addressed explicitly using mixed-effects models controlling for individual.

We fitted GAM models using the mgcv package in R (https://CRAN.R-project.org/package=mgcv) with the response variables of each dimension (length, width or depth), plus the overall bite volume (length×width×depth) and independent covariates of species and strike type, and independent continuous kinematic variables of peak gape, peak jaw protrusion, peak lower jaw angle, time to peak gape and ram speed. We did not assess the effect of kinematics on surface area (width×length) because bite width was highly invariant across an individual's strikes, indicating its tight correlation with a fish's nearly fixed gape width, whereas length (and depth) were highly variable across an individual's strikes. Thus, we wanted to evaluate the effects of kinematics on bite length independently of gape width.

We used the REML method for calculating smoothness of splines and Gaussian distributions for all models. We compared the fit of each kinematic variable modeled as either a linear term or a smoothing spline using model selection with the AIC criterion in R. We further allowed for shrinkage of each smoothing spline within the full model to determine which kinematic variables were best modeled as spline terms. We then systematically compared models with both two-way thin-plate splines or all one-way splines to explore whether there were any non-linear interactions between kinematic variables. Model fits were visualized with the mgViz (Fasiolo et al., 2020) and ggplot2 packages (https://CRAN.R-project.org/package=ggplot2) in R. All coding was assisted by suggestions from ChatGPT 3.5 and 4.0 (OpenAI, Inc.). R code and raw data for all kinematic calculations and analyses is provided in Datasets 1–5.

## RESULTS

### Scale eaters display increased gel bite length

**Fig. 3.**
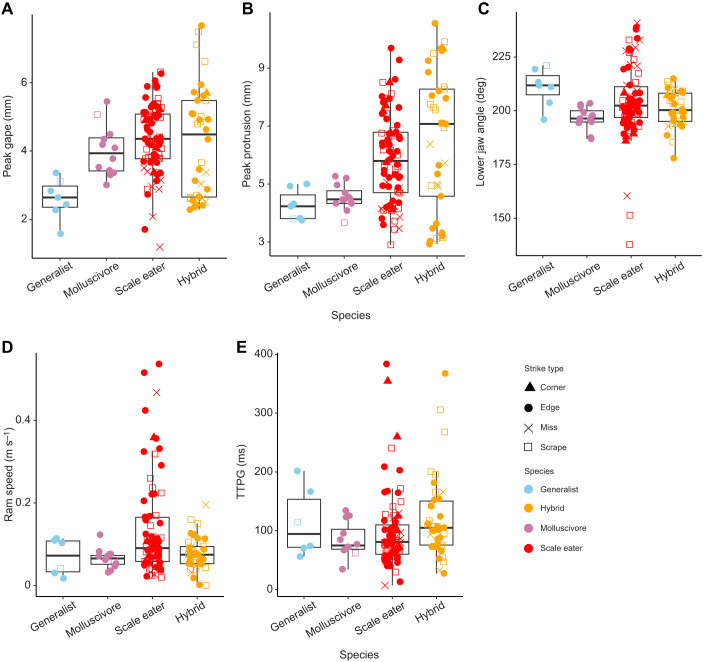
**Five key kinematic variables do not significantly vary across species or strike type during gel-biting strikes.** Boxplots overlaid with raw data show (A) peak gape, (B) peak protrusion, (C) lower jaw angle, (D) ram speed and (E) time to peak gape (TTPG) measured during gel-biting strikes from automated landmarking of 227 videos. Lower jaw angle is the minimum angle between the lower jaw and suspensorium from 20% to peak gape. Ram speed was calculated from the distance traveled between 20% and peak gape. Species or hybrid cross is indicated by symbol color and strike type is indicated by symbol shape.

In mixed-effects models controlling for species, strike type and repeated sampling of each individual, we found significant effects of species on gel cube bite length (Fig. 2; *P*=0.0007, type II ANOVA) and width (*P*=0.049, type II ANOVA), but not depth (*P*=0.289, type II ANOVA). However, only scale eater versus generalist *post hoc* contrasts were significantly different in bite length (*P*=0.028, Tukey *post hoc* test) and there were no significant *post hoc* pairwise contrasts for bite width or depth. Furthermore, using mixed-effects models controlling for repeated sampling of individuals, we found that species and strike type were not significantly associated with any of the five kinematic variables in *post hoc* comparisons or overall via type II ANOVA, even when comparing missed strikes with successful bites (Fig. 3).

### Substantial similarity in strike kinematics among species and strike types

**Fig. 4.**
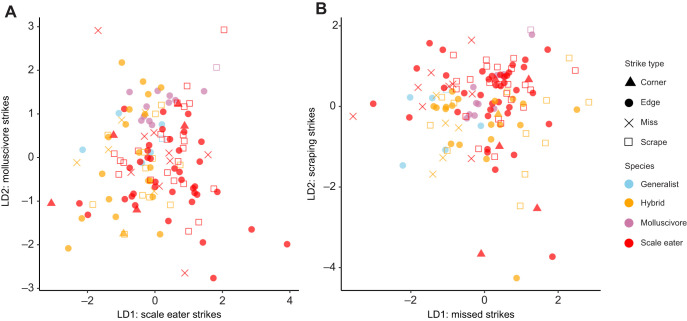
**Linear discriminate analyses illustrate substantial overlap by strike type and species.** (A) Discriminant analysis by species. (B) Discriminant analysis by strike type. Species or hybrid cross is indicated by symbol color and strike type is indicated by symbol shape. Multivariate analyses were based on five kinematic variables: peak gape, peak jaw protrusion, minimum lower jaw angle with the suspensorium, TTPG and ram speed.

Linear discriminant analysis by strike type successfully classified strikes at a rate of only 50.8% (Fig. 4; 25% would be random classification at four factor levels). The kinematic variable best separating misses from other strike types on discriminant axis one was peak gape. Linear discriminant analysis by species successfully classified species or hybrids based on their strike kinematics at a rate of only 32.3% (25% would be random classification at four factor levels). The kinematic variables best separating scale eaters from other groups on discriminant axis one (Fig. 4) were peak gape again and peak jaw protrusion, while TTPG had the weakest effect on classification of species by kinematic variables. Although plots of the first two discriminant axes indicate greater variation within scale eater and hybrid strike kinematics, there was also clearly substantial overlap among species and hybrids (Fig. 4).

### Multi-peak performance landscape for gel biting

**Fig. 5.**
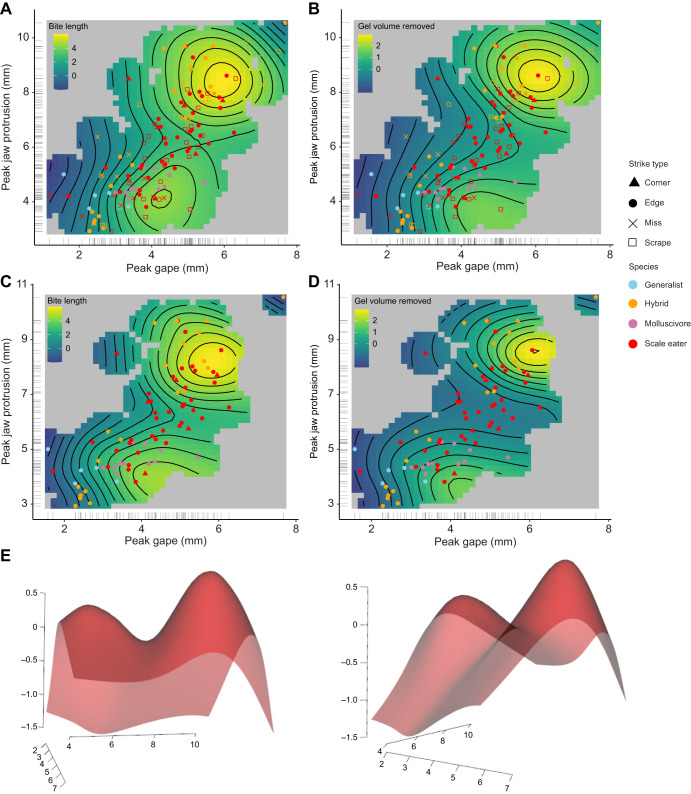
**Generalized additive modeling supports a non-linear interaction between peak gape and peak protrusion for bite performance.** Thin-plate splines from the best-fitting GAM model (Table 1) for the response variable of bite length (A,C) or total bite size (gel volume removed: B,D). The first row includes all strike types and the second row excludes scraping and missed strikes. Species or hybrid cross is indicated by symbol color and strike type is indicated by symbol shape for 130 strikes with data for gel bite dimensions. Gel volume removed was calculated from length×width×depth of each gelatin bite measured with digital calipers under a stereomicroscope. (E) Performance landscape for bite length (mm) in 3D perspective view.

We used generalized additive modeling to explore the relationship between kinematic variables and the response variables of total bite volume removed per strike and each bite size dimension separately (length, width and depth). The best fitting model for bite volume (Table 2) included a 2D thin-plate spline for peak gape and peak jaw protrusion along with fixed linear predictors for species, strike type, peak lower jaw angle, TTPG and ram speed. The non-linear interaction between peak gape and peak jaw protrusion was significantly associated with both the bite length (estimated degrees of freedom, edf=10.82, *P*=9e−7) and the overall gel volume removed (edf=8, *P*=0.0008) and displayed a bimodal surface with two isolated performance peaks (Fig. 5). The best-fit model for bite length included additional significant linear effects of ram speed (*P*=0.008) and peak lower jaw angle (*P*=0.007), but not TTPG (*P*=0.440) in addition to significant factor levels of missed strikes (*P*=1.12e−10) and molluscivore species (*P*=0.012). Models without TTPG fitted the data equally well (ΔAIC<2). Even after excluding missed strikes that made contact but left no mark on the gel (Fig. 1) and scraping bites in which the jaws did not fully occlude, the interaction between peak gape and jaw protrusion was still significantly associated with edge and corner bite length (edf=10.34, *P*=9e−6) and volume (edf=11.17, *P*=4.89e−5). Strikes by different species were not restricted to a single performance optimum and in many cases exhibited substantial kinematic strike variation. For example, strikes by scale eaters were observed on both performance peaks, the intermediate performance valley between them, and surrounding low performance regions (Fig. 5).

**Table 2.**
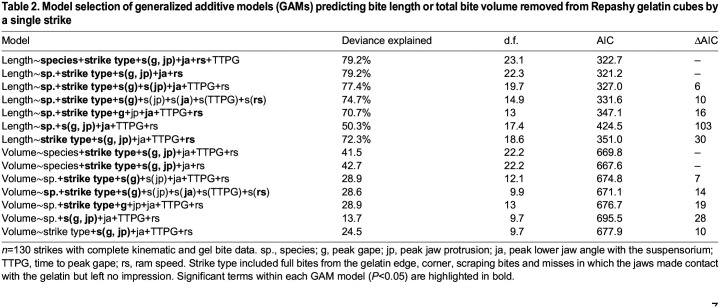
Model selection of generalized additive models (GAMs) predicting bite length or total bite volume removed from Repashy gelatin cubes by a single strike

Bite width was significantly linearly associated with the interaction between peak gape and peak jaw protrusion, resulting in a flat performance landscape (edf=1.759, *P*=0.0002), which is largely controlled by the morphological dimensions of the oral jaws of each fish, rather than kinematic variables. Bite depth was not significantly associated with peak gape and peak jaw protrusion, or with any independent variable in this model except for strike type, as expected because scraping strikes are extremely shallow by definition (*P*=0.046).

## DISCUSSION

We estimated a bimodal non-linear performance landscape for the unusual trophic niche of lepidophagy from high-speed videos of gelatin-removing bites. In contrast to studies of suction-feeding performance on evasive, attached, and strain-sensitive (e.g. zooplankton) prey (Holzman et al., 2008, 2022; Olsson et al., 2020), we found no effect of kinematic timing variables such as TTPG or even ram speed on the performance of scale-biting strikes, measured by the length, width, depth and total volume of gelatin removed. Instead, successful scale biting appears to require strike coordination between jaw opening (peak gape) and jaw protrusion and, surprisingly, this interaction resulted in two distinct performance optima: (1) strikes with small peak gapes removed the greatest amount of material per bite at small jaw protrusion distances; (2) strikes with large peak gapes removed the greatest volumes at large jaw protrusion distances. These two performance optima were surrounded by reduced bite performance in all directions including more extreme values and intermediate values of peak gape and protrusion. This resulted in two distinct performance peaks on the 2D thin-plate spline for jaw protrusion and peak gape in the model best supported by the data (Fig. 4, Table 2). The largest and smallest peak gapes and jaw protrusion distances observed across all strikes still suffered a performance decline. This falloff of bite performance at maximum and minimum peak gapes and jaw protrusion distances is consistent with muscle length–tension curves (Williams et al., 2013), but does not explain the fall off in performance at intermediate distances (the performance valley between the two optima) when muscle force should be highest. Thus, we found evidence of a two-peak performance landscape for the functional task of biting, a well-studied behavior across vertebrates which is often viewed as a simple mechanical system (Herrel et al., 2008; Wainwright and Richard, 1995; Westneat, 2005; but see studies of bite force dynamics in a field setting: Gidmark et al., 2013; Perevolotsky et al., 2020; Shimada et al., 2012).

### Two distinct performance optima for biting rather than a linear ridge

We expected to observe a simple linear ridge for the interaction between peak gape and peak jaw protrusion in relation to bite volume and bite length based on muscle length tension curves, biomechanical models of bite force and our previous work (St John et al., 2020a,b; Westneat, 2003; Williams et al., 2013). Instead, both extreme and intermediate values of these kinematic variables resulted in poor bite performance (Fig. 5). Some of the strikes performed by scale-eating specialists and their hybrids resided on the highest performance peak observed, requiring larger peak gape and jaw protrusion distance, while all strikes by generalists, molluscivores and their hybrids were restricted to the region containing a different performance peak for smaller gape and jaw protrusion distances. These performance optima were separated by a valley, isolating the recently evolved scale-eating specialist *C. desquamator* and its hybrids from its generalist ancestor. However, substantial individual strike variation was observed across all species, such that kinematic variables were unable to distinguish among species or among the different strike types of scraping, corner, edge or completely missed strikes (Figs 3–5). Scale eater strikes exhibited almost the entire range of kinematic variation observed, spanning both performance optima and valleys surrounding them; however, only scale eaters and their hybrids were able to perform strikes within the kinematic range of the highest performance peak observed (Fig. 5).

Explanations for this performance landscape must also account for the poor performance of the intermediate strike values observed, rather than the performance ridge that we expected based on the known importance of strike coordination between peak gape and peak jaw protrusion (Holzman et al., 2007; Wainwright et al., 2008). One possible explanation is a biomechanical tradeoff in precision and targeted bite area, with the most adverse effects on gel-biting performance at intermediate values. Interactions between oral jaw scraping and biting with the gelatin surface may only be effective within two different kinematic regimes. Smaller peak gapes with less jaw protrusion may allow for more precise targeting of the gel edge for efficient bites with greater force. Larger peak gapes with greater jaw protrusion may reduce precision and bite force, but cover a larger area, resulting in more gelatin removed per bite. Intermediate values may suffer the costs of less precise biting and less area covered per bite. In the field, this tradeoff may allow for more precisely targeting particular regions of elusive prey, such as the lower flank where scales are more easily removed, or large, scraping bites with the jaws extended to remove as much material as possible with low precision. It is tempting to speculate that strike speed or lower jaw angle play a role in this precision/target area tradeoff. However, while ram speed and lower jaw angle with the suspensorium both had strong linear effects on bite performance, there was no evidence of any non-linear interactions with peak gape or peak jaw protrusion distance (Table 2). Similarly, timing (TTPG) seems to play no role in bite performance, which would seem surprising if precision is important for gelatin removal because faster time to peak gape should reduce bite precision. However, biting strikes generally achieved peak gape substantially before contact with the gel, suggesting that the time to reach peak gape is not important as long as the jaw is open at some point before contact with the target. This is consistent with the ‘plateau effect’ observed in the scale-eating piranha, the only other direct study of scale-biting kinematics in other lepidophagous species [Janovetz, 2005; but see behavioral laterality studies of *Perissodus microlepis* (Hori, 1993; Hori et al., 2007; Kovac et al., 2019; Lee et al., 2015; Takeuchi et al., 2012) and *Exodon paradoxus* (Hata et al., 2011)].

A second possible explanation for the two-peak performance landscape is the striking observation that none of the generalist or molluscivore species exhibited feeding strikes with jaw protrusion distances within range of the second, higher performance peak (Fig. 5). Conversely, only scale eaters and hybrids with scale eater ancestry were capable of producing feeding strikes with jaw protrusion distances in this range (>6.5 mm; see Dataset 3; Fig. 5). Thus, there appears to be a genetic basis underlying the two performance peaks: only scale eaters and hybrids with scale eater ancestry can protrude their jaws sufficiently to reach the second performance optimum. This may be due to additional anatomical properties of their oral jaws that allow for greater extension during strikes, such as different ratios of muscle fiber types within the adductor mandibulae (Ono and Kaufman, 1983; Summers and Long, 2005), along with unmeasured aspects of their behavior or strike kinematics. Indeed, scale eaters exhibit significant differences in their boldness and exploratory behaviors (St John et al., 2020a,b). Genome-wide association scans for oral jaw length in this radiation identified collagen genes with fixed regulatory differences between scale eaters and molluscivores, such as collagen type XV alpha 1 (col15a1), suggesting that the elasticity of jaw opening may be under selection in this species (Martin et al., 2019; McGirr and Martin, 2017). Greater peak gapes are possible because of the twofold larger oral jaws of the scale eater, based on our previous skeletal measurements of these species (Hernandez et al., 2018; Martin, 2016b). However, scale eaters still do not open their jaws as wide as possible during strikes or achieve 180 deg angles with their open jaws as in other scale-eating specialists such as the scale-eating piranha (Janovetz, 2005), indicating adaptive behavioral compensation for their extreme oral anatomy during strikes (St John et al., 2020a,b).

Finally, we cannot rule out more esoteric explanations for the unexpected fitness valley between bite performance optima. Sensory perception during strikes may be limited at intermediate strike distances as a result of the blind spot caused by the positioning of the vertebrate optic nerve in front of the retina, although the biomechanical implications of this in fishes are unknown (Gregory and Cavanagh, 2011). Alternatively, intermediate jaw protrusion may be an indirect effect of premature suspension of strike behavior or lack of motivation during the strike. However, excluding missed strikes did not alter the observed two-peak performance landscape (Fig. 5).

### Similarity between the performance and fitness landscapes

Both field measurements of fitness and laboratory measurements of scale-biting performance support a two-peak landscape, although these landscapes do not directly align and there is substantial many-to-many mapping between species morphology and performance. This is the first study providing any support for increased fitness of scale eater specialists, in this case through a second, higher performance peak accessible only to scale eaters and their hybrids. Repeated field experiments on San Salvador Island measured two-peak fitness landscapes from the growth and survival of advanced generation hybrids placed within field enclosures in their natural hypersaline lake environments for 3–11 months (Martin and Wainwright, 2013b). These landscapes remained relatively stable and exhibited a similar two-peak topography across years, lakes and frequency manipulations of hybrids (Martin, 2016a; Martin and Gould, 2020). However, in all cases where it was detected, the first and second peaks corresponded to the morphology of generalists and molluscivores, respectively; whereas hybrids resembling the scale eater survived and grew at the lowest rates in all field fitness experiments. Thus, we have evidence for a single fitness peak corresponding to hybrids with generalist morphology (Martin and Gould, 2020; Martin and Wainwright, 2013b) and a single performance peak for the strike kinematics of both generalist and molluscivore species (this study). However, the higher scale-biting performance peak in this study has no analog in any field enclosure experiments, perhaps because of a lack of hybrids with the necessary combination of scale-biting behavior, physiology and craniofacial morphology. Lab-reared F2 hybrids with scale eater ancestry displayed sufficient jaw protrusion distance during gel-biting strikes to occupy the second performance peak so it is still unclear why no fitness peak for scale eater phenotypes has yet been detected in the wild.

Our results may also indicate that these laboratory gel-biting performance trials do not reflect a functional task relevant to the survival and growth rate of scale eaters in the wild. Admittedly, biting gels may exert different functional demands from scraping scales and mucus from the sites of a moving fish. However, previous videos of both gel-biting and scale-scraping strikes on euthanized zebrafish indicated similar strike kinematics on the two prey types (St John et al., 2020a,b). We think it is more likely that the hybrid phenotypes tested in field enclosures were unable to efficiently perform scale-eating strikes in order to survive and grow over the course of the field experiments or that the environment inside the field enclosures limited the viability of scale eating as a potential trophic niche, perhaps because of increased opportunities for prey learning within a small captive population (Hori, 1993; Martin and Gould, 2020). Periodic resource availability bottlenecks may also increase the long-time reproductive success of scale eaters in the wild (Liem, 1980; Galvez et al., 2022; Martin and Genner, 2009; Robinson and Wilson, 1998), although so far scale-eating strikes have been observed during every year and season on the island.

Ultimately, the goal of connecting morphological fitness landscapes to performance landscapes is difficult because of many-to-many mapping and the curse of dimensionality (Bergmann and Tonelli-Sippel, 2024). We observed no clear mapping of species morphology onto strike kinematics and observed substantial variation in strike kinematics within each individual, sometimes spanning nearly the entire performance landscape for scale eaters and their hybrids (Figs 3–5). However, a better understanding of the genetic basis of kinematic variables, such as gape and jaw protrusion distance, may enable future studies to connect the genetic regulatory networks underlying morphological, kinematic and behavioral traits quantified in the lab to genotypic fitness networks measured from field fitness experiments. Our initial study of genotypic fitness networks found that 2% of pathways through genotype space connected generalist to scale eater haplotypes through a series of intermediate steps along the network known to be equal or increasing in fitness based on observed hybrid fitness from field experiments (Patton et al., 2022). However, connecting these genotypes and phenotypes to gel-biting performance is formidable because a single individual can sometimes perform nearly the entire range of kinematic variation observed across all species in this study. This immense many-to-many mapping of morphology to performance suggests that experimental genomic manipulation of single genes or variants may allow us to precisely quantify the effect sizes propagating from genotype to phenotype to performance to fitness landscape as Arnold (1983) originally proposed. However, it is unclear how quickly these effects will attenuate across levels of biological organization.

### Conclusion

Here, we explored the biomechanics of a highly specialized trophic niche and demonstrate the power of machine-learning approaches to automate landmarking of kinematic data. We estimated a surprisingly complex two-peak performance landscape for biting that indicates that the highly protrusible jaws of scale-eating specialists may provide a performance benefit for scale eating. This study provides a new framework for understanding bite mechanics in fishes and a foundation for dissecting the genetic basis of specialized predatory niches and their relationship to fitness landscapes driving rapid adaptive radiation in the wild.

## Supplementary Material

10.1242/jexbio.247615_sup1 Supplementary information

Dataset 1. raw .hd5 output files from SLEAP inference pipeline for all strike videos.

Dataset 2. Kinematic data output from SLEAP-kinematics-data.extraction.R script with SLEAP output files as the input.

Dataset 3. Kinematic and bite dimensional data used for all figures and statistical analyses in the manuscript.

Dataset 4. SLEAP-kinematics-data.extraction.R script R script for extracting coordinates from .hd5 files output by SLEAP inference pipeline and then calculating kinematic variables for each strike sequence.

Dataset 5. analyses.R R script for generating all figures and performing all statistical analyses in the manuscript.
