# Supplementary material for: A multi-peak performance landscape for scale biting in an adaptive radiation of pupfishes
Source: J Exp Biol. 2024 Aug 23;227(16):jeb247615. doi: 10.1242/jeb.247615 (PMC11418179; doi:10.1242/jeb.247615)
Supplement: Supplementary information [file jexbio-227-247615-s1.pdf]

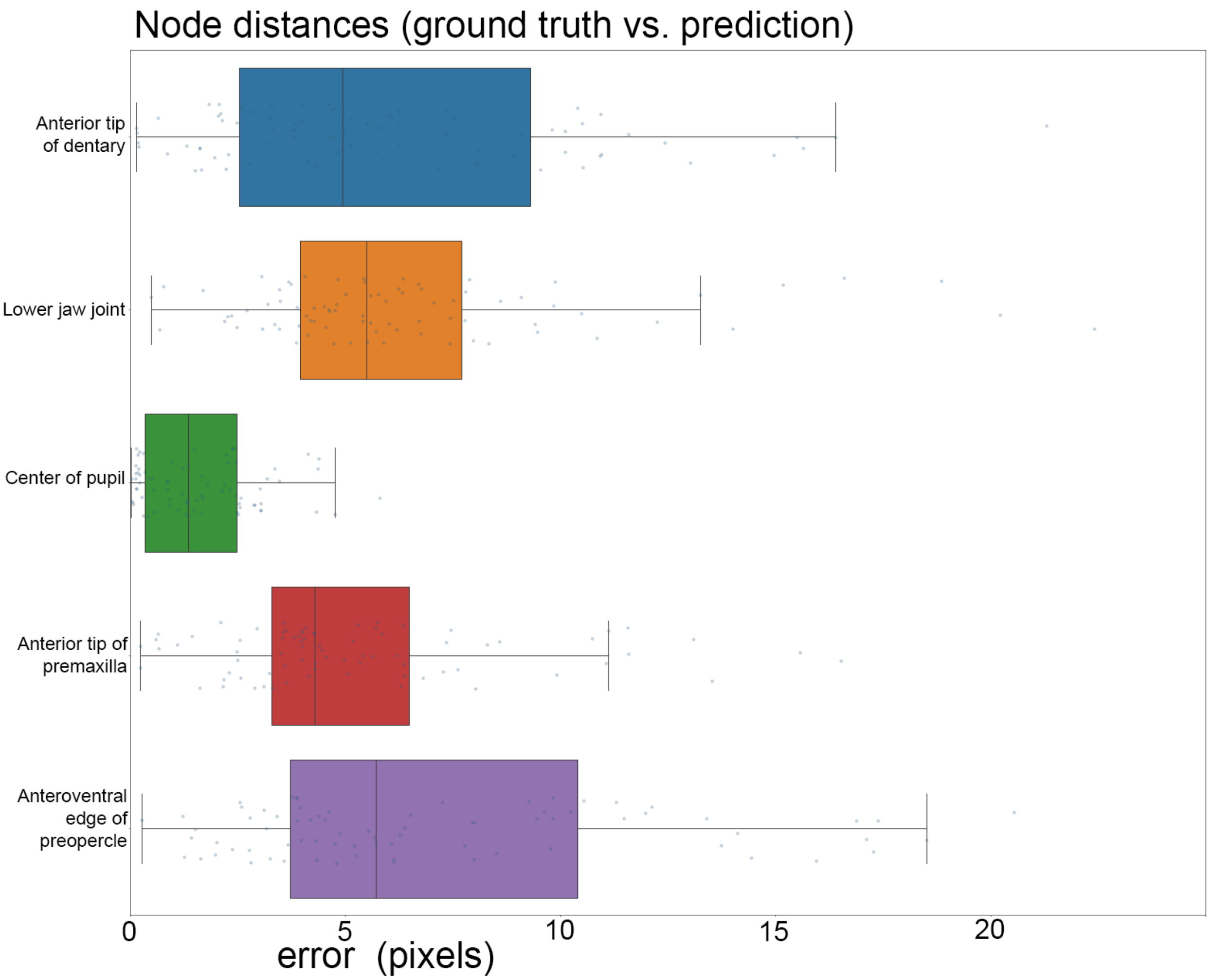

Fig. S1. Estimated error from the trained SLEAP model used for inferences in this study. We achieved the greatest number of landmarked videos and frames using the multi-animal bottom-up unet model with a receptive field of 156 pixels, max stride of 32 pixels, batch size of 3, input scaling of 0.75, and validation fraction of 0.1.

Journal of Experimental Biology • Supplementary information

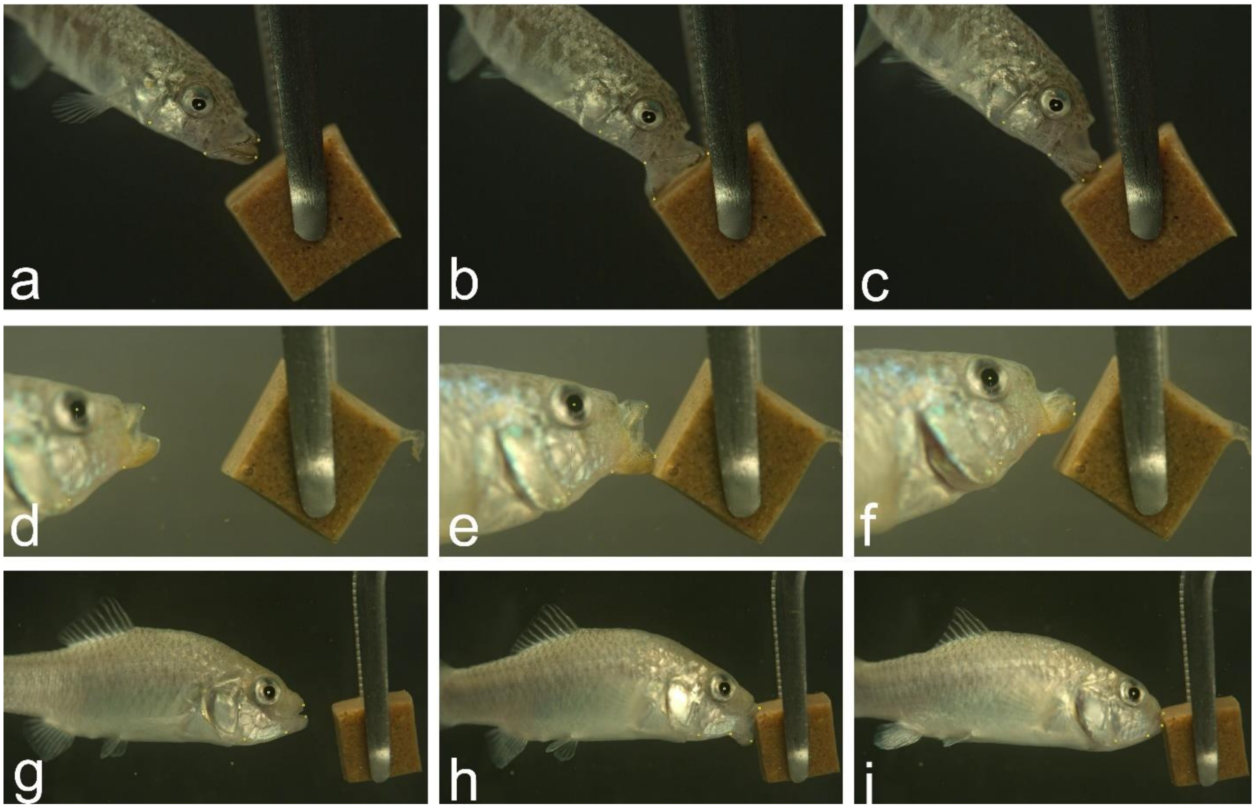

Fig. S2. Digitizing high-speed video of gel-biting strikes with automated landmarking software. Strike sequences for a scraping bite by a scale-eater (a-c), a miss by a hybrid (d-f), and an edge bite by a molluscivore (g-i). Videos were filmed at 1,100 fps on a Phantom VEO 440S camera. Frames illustrate approximately 20% of peak gape (first column), peak gape (second column), and jaw adduction immediately after the bite (third column). Five landmarks on each frame were placed automatically using our SLEAP inference model and are illustrated as small yellow dots to emphasize the accuracy of these inferred landmarks.

Journal of Experimental Biology • Supplementary information

Dataset 1. raw .hd5 output files from SLEAP inference pipeline for all strike videos.

Available for download at
https://journals.biologists.com/jeb/article-lookup/doi/10.1242/jeb.247615#supplementary-data

Dataset 2. Kinematic data output from SLEAP-kinematics-data.extraction.R script with SLEAP output files as the input.

Available for download at
https://journals.biologists.com/jeb/article-lookup/doi/10.1242/jeb.247615#supplementary-data

Dataset 3. Kinematic and bite dimensional data used for all figures and statistical analyses in the manuscript.

Available for download at
https://journals.biologists.com/jeb/article-lookup/doi/10.1242/jeb.247615#supplementary-data

Dataset 4. SLEAP-kinematics-data.extraction.R script

R script for extracting coordinates from .hd5 files output by SLEAP inference pipeline and then calculating kinematic variables for each strike sequence.

Available for download at
https://journals.biologists.com/jeb/article-lookup/doi/10.1242/jeb.247615#supplementary-data

Dataset 5. analyses.R

R script for generating all figures and performing all statistical analyses in the manuscript.

Available for download at
https://journals.biologists.com/jeb/article-lookup/doi/10.1242/jeb.247615#supplementary-data

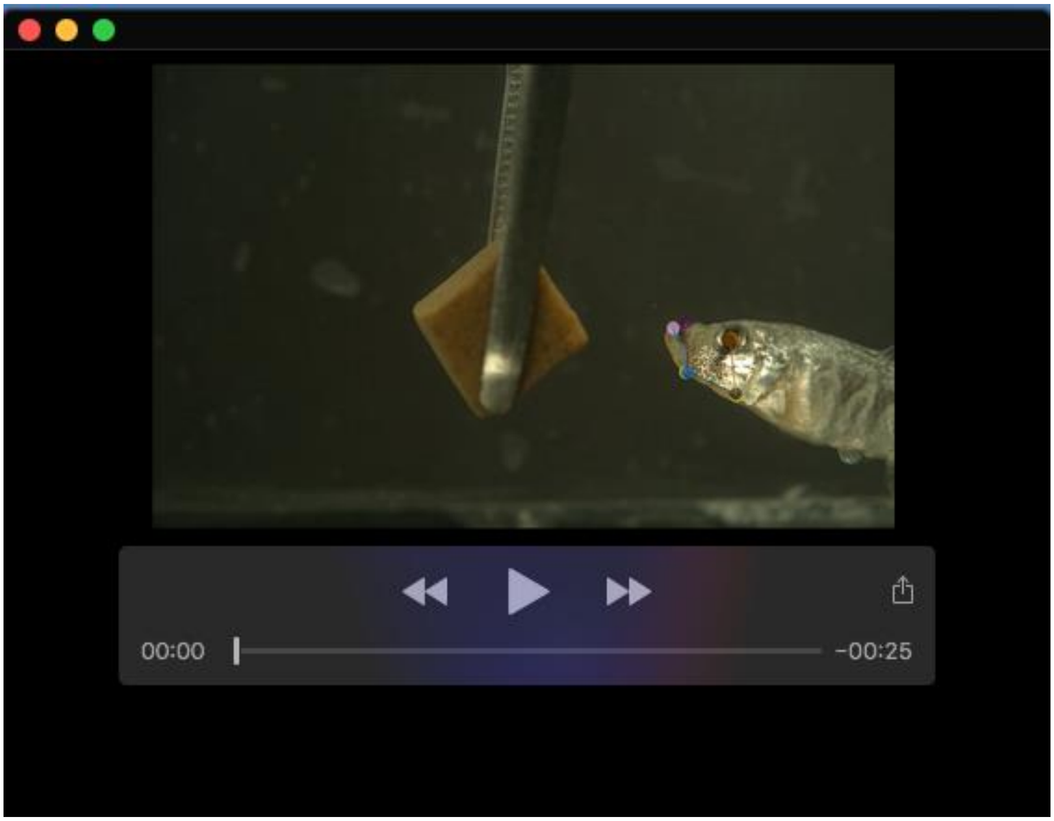

Movie 1. High-speed video of gel-biting strikes by a scale-eater with automated landmarks. Videos were filmed at 1,100 fps on a Phantom VEO 440S camera. Yellow landmarks on each frame were placed automatically using our SLEAP inference model. Human-placed labels that were part of the training set periodically appear as colored landmarks.

Journal of Experimental Biology • Supplementary information

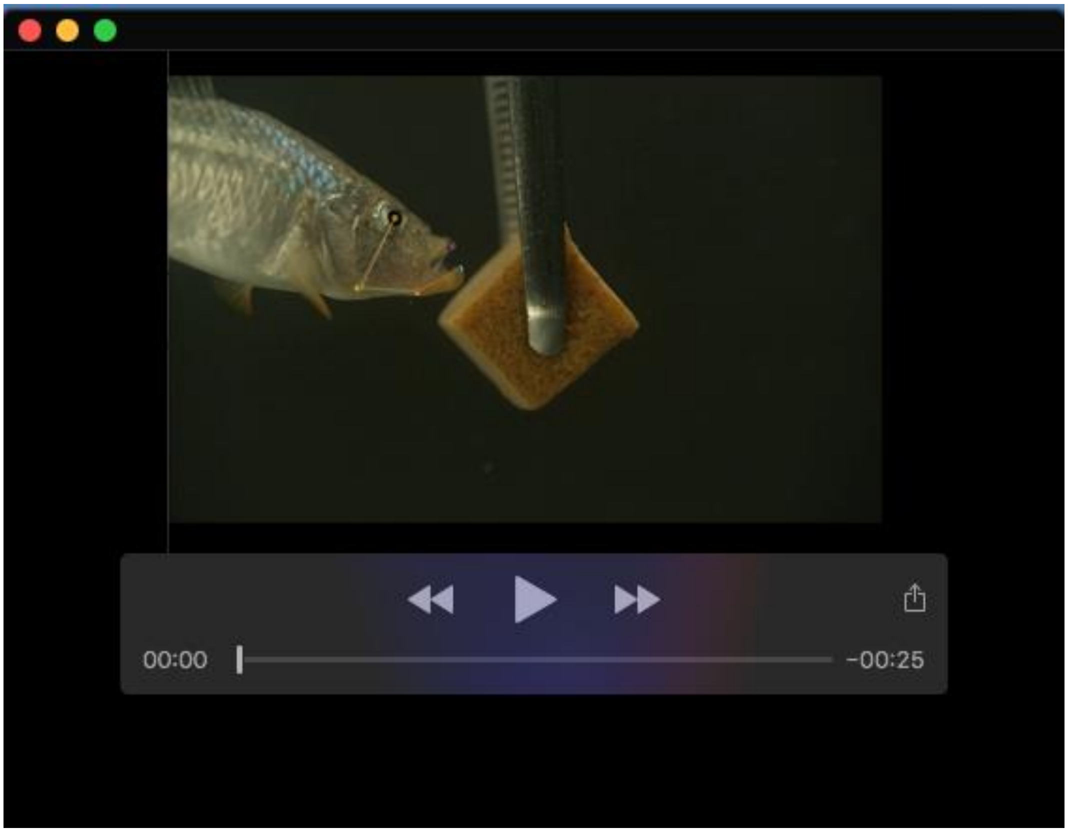

Movie 2. High-speed video of gel-biting strikes by another scale-eater with automated landmarks. Videos were filmed at 1,100 fps on a Phantom VEO 440S camera. Yellow landmarks on each frame were placed automatically using our SLEAP inference model. Human-placed labels that were part of the training set periodically appear as colored landmarks.

Journal of Experimental Biology • Supplementary information

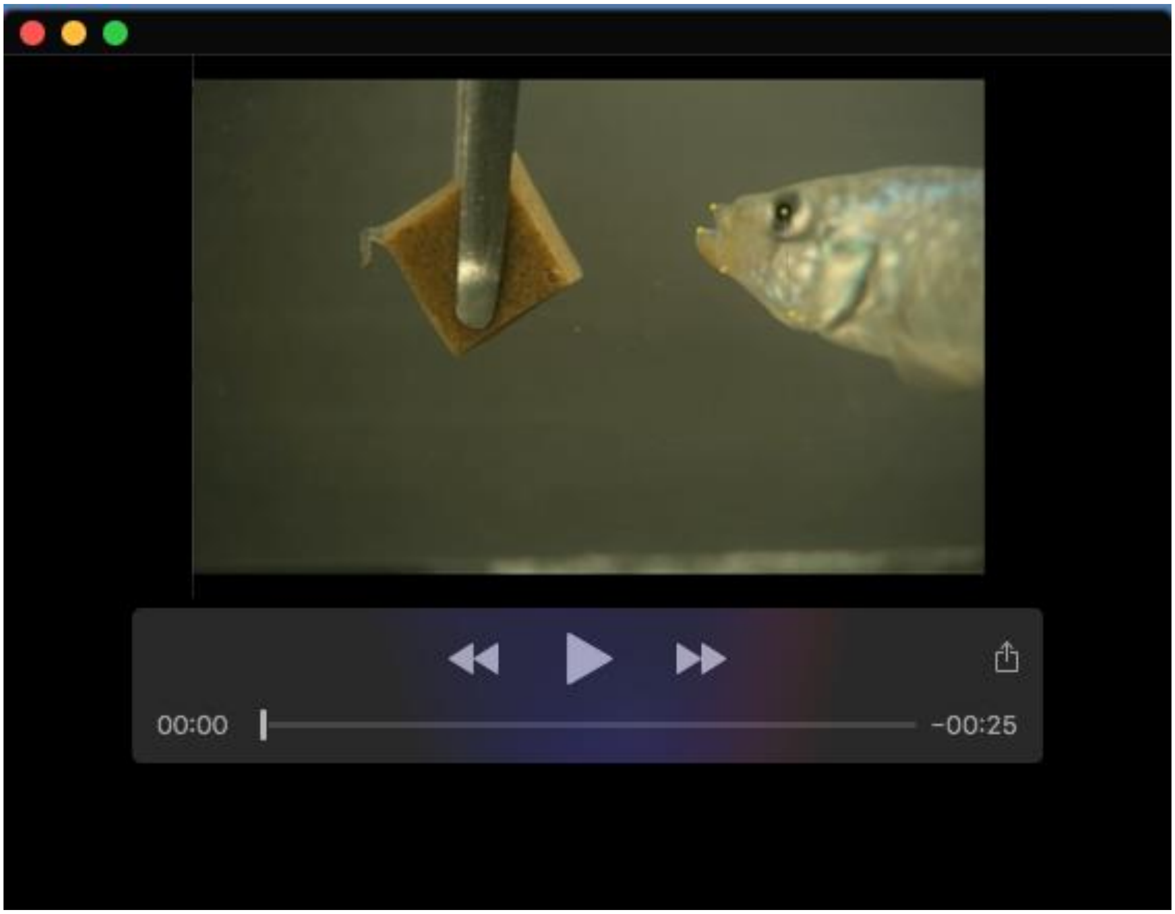

Movie 3. High-speed video of a gel-biting missed strike by a hybrid with automated landmarks. Videos were filmed at 1,100 fps on a Phantom VEO 440S camera. Yellow landmarks on each frame were placed automatically using our SLEAP inference model. Human-placed labels that were part of the training set periodically appear as colored landmarks.

Journal of Experimental Biology • Supplementary information
